# Epidemiology of Sexually Transmitted Diseases Among Pregnant Adolescents

**DOI:** 10.1155/S1064744994000128

**Published:** 1994

**Authors:** Lisa N. Gittens, Rhonda R. Nichols, Joseph J. Apuzzio

**Affiliations:** 1 Department of Obstetrics and Gynecology New Jersey Medical School Newark NJ USA; 2 Department of Obstetrics and Gynecology New Jersey Medical School 185 South Orange Avenue E506 Newark NJ 07103 USA

## Abstract

*Objective:* The purpose of this study was to determine the epidemiology of sexually transmitted diseases (STDs) among pregnant adolescents.

*Methods:* Charts of all patients (n = 735) who attended the Maternal and Infant Care Clinic at University Hospital, Newark, NJ, between July 1, 1991, and June 30, 1992, were reviewed for STDs which included gonorrhea, chlamydia, syphilis, and human immunodeficiency virus (HIV). At the first prenatal visit, each registrant had endocervical specimens obtained to detect gonorrhea and chlamydia. A serum sample was obtained for syphilis screening. HIV testing was made available to all patients and testing was done on a voluntary basis. The same STD screening that was done at the initial visit was repeated at 28 and 36 weeks.

*Results:* Twenty-five percent of patients tested positive for one or more STDs. The mean patient age was 17.3 years. The mean gestational age at first visit was 19.5 weeks. The mean number of visits was 7.3. The following STDs were identified: 4.8% of patients tested positive for gonorrhea, 20.9% tested positive for chlamydia, and 1.7% tested positive for syphilis. Twenty-one percent of patients had a positive STD diagnosed at the initial visit. Another 4.8% of patients had an STD diagnosed at some time after the initial visit when the initial screen was negative for STDs. An additional 1% of patients who initially tested positive for an STD had subsequent screening which revealed another STD (different organism). Seven patients tested HIV positive. Sixty-one percent of patients with STDs agreed to HIV testing. One patient had HIV coexistent with another STD.

*Conclusions:* Pregnant adolescents are at risk for multiple STDs. HIV testing should be offered. STD screening should be repeated in the third trimester in adolescent patients.


Infectious Diseases in Obstetrics and Gynecology 1:216–219 (1994)
© 1994 Wiley-Liss, Inc.

### Epidemiology of Sexually Transmitted Diseases Among Pregnant Adolescents

**Lisa N. Gittens, Rhonda R. Nichols, and Joseph J. Apuzzio**
*Department of Obstetrics and Gynecology, New Jersey Medical School, Newark, NJ*

### Abstract

***Objective:*** The purpose of this study was to determine the epidemiology of sexually transmitted diseases (STDs) among pregnant adolescents.

***Methods:*** Charts of all patients (n = 735) who attended the Maternal and Infant Care Clinic at University Hospital, Newark, NJ, between July 1, 1991, and June 30, 1992, were reviewed for STDs which included gonorrhea, chlamydia, syphilis, and human immunodeficiency virus (HIV). At the first prenatal visit, each registrant had endocervical specimens obtained to detect gonorrhea and chlamydia. A serum sample was obtained for syphilis screening. HIV testing was made available to all patients and testing was done on a voluntary basis. The same STD screening that was done at the initial visit was repeated at 28 and 36 weeks.

***Results:*** Twenty-five percent of patients tested positive for one or more STDs. The mean patient age was 17.3 years. The mean gestational age at first visit was 19.5 weeks. The mean number of visits was 7.3. The following STDs were identified: 4.8% of patients tested positive for gonorrhea, 20.9% tested positive for chlamydia, and 1.7% tested positive for syphilis. Twenty-one percent of patients had a positive STD diagnosed at the initial visit. Another 4.8% of patients had an STD diagnosed at some time after the initial visit when the initial screen was negative for STDs. An additional 1% of patients who initially tested positive for an STD had subsequent screening which revealed another STD (different organism). Seven patients tested HIV positive. Sixty-one percent of patients with STDs agreed to HIV testing. One patient had HIV coexistent with another STD.

***Conclusions:*** Pregnant adolescents are at risk for multiple STDs. HIV testing should be offered. STD screening should be repeated in the third trimester in adolescent patients.
© 1994 Wiley-Liss, Inc.

Key words
Pregnancy, chlamydia, gonorrhea, HIV

The number of sexually active adolescents has risen dramatically in the past 15 years. Studies in 1988 show that 25% of 15-year-old girls and 75% of 19-year-old girls have had sexual intercourse.[1,2] An estimated 1,014,680 teens became pregnant in 1987, with 50% of these pregnancies resulting in births. The teenage birth rate in 1989 (girls aged 15–19 years) was 49/1,000 among whites and 97/1,000 among minorities; the teenage birth rate in the United States is higher than in many developing countries.[2,3]

Pregnancies among adolescents, especially those under age 15 years, are complicated by adverse neonatal outcome.[4,5] Research has shown, however, that maternal age alone has little effect on poor outcome when other contributing risk factors such as inadequate maternal weight gain, poor nutrition, and substance abuse are controlled for. The presence of a sexually transmitted disease (STD) represents an additional but correctable risk for the pregnant adolescent. Infections with herpes, syphillis, gonorrhea, and chlamydia may cause disease result-

Address correspondence/reprint requests to Dr. Joseph Apuzzio, Department of Obstetrics and Gynecology, New Jersey Medical School, 185 South Orange Avenue, E506, Newark, NJ 07103.

Received September 15, 1993

Accepted January 24, 1994

Clinical Study ing in preterm labor, chorioamnionitis, and sepsis in the mother and pneumonia, conjunctivitis, congenital malformation, and sepsis in the neonate.[6,7] The range of perinatal morbidity associated with human immunodeficiency virus (HIV) infection is still being described. Depending upon the population studied, 8–25% of sexually active adolescents have endocervical infections with *Chlamydia trachomatis* and 0.4–12% have endocervical infection with *Neisseria gonorrhoeae*.[8–11] A 0.7% incidence rate of syphilis among a group of 15–19-year-old indigent pregnant girls has been reported.[12] As of March 1990, 1,429 cases of acquired immunodeficiency syndrome (AIDS) in adolescents have been reported to the Centers for Disease Control and Prevention. There are no studies that review the epidemiology of STDs including HIV in the adolescent pregnant patient. To aid in targeting sites for intervention, we studied the epidemiology of STDs in an inner-city pregnant adolescent population.

### MATERIALS AND METHODS

All patients who had at least one prenatal visit to the Maternal and Infant Care Clinic at University Hospital, Newark, NJ, and delivered at one institution between July 1, 1991, and June 30, 1992, were included in the study. As part of routine prenatal care, all patients were screened for *N. gonorrhoeae, C. trachomatis,* and syphilis at the first visit. Two endocervical specimens were obtained. The first was inoculated on Thayer Martin medium, incubated, and studied for the presence of *N. gonorrhoeae;* the second was processed for chlamydia detection by immunoassay (Chlamydiazyme, Abbott Laboratories, Chicago, IL). A serum sample was obtained for Rapid Plasma Reagin (RPR). Fluorescent treponemal antibody testing (FTA) was performed on blood from all patients who tested RPR positive. The above-described STD screening was repeated at 28 and 36 weeks gestation.

All patients with suspicious genital lesions had cultures for herpes simplex virus. At the first prenatal visit, patients were counseled regarding HIV. Voluntary HIV screening by enzyme-linked immunosorbent assay (ELISA) (Recombigen, Cambridge Biotech Corp., Worchester, MA) was performed on all patients who agreed to testing. A positive culture for gonorrhea or a positive Chlamydiazyme was considered diagnostic of the respective disease. Patients were described as having syphilis if the RPR and FTA were positive (patients with history of infection and positive titers were excluded from this group). All patients who tested HIV positive by ELISA had HIV confirmation by Western blot analysis (Novapath, Bio-Rad Laboratories, Hercules, CA). Results of STD screening and demographic information regarding the patient's age, parity, gestational age at time of first visit, and the number of prenatal visits were recorded.

Demographic data were reviewed to determine measures of central tendency and proportions that described the patient population. Results of STD screening were reviewed to determine percentages of disease in our population. Means and ranges were used where appropriate.

### RESULTS

### Maternal Characteristics

A total of 735 adolescent patients enrolled in our clinic delivered between July 1, 1991, and June 30, 1992. Our patients were predominantly black and Hispanic females. Of the 735 enrolled patients, 188 patients (25.2%) had an STD. The mean age for patients with STDs was 17.3 years (range 12–20 years). The mean number of prenatal visits was 7.3 (range 1–13 visits). The mean gestational age at the time of the first visit was 19.5 weeks (range 6–35 weeks); 57 patients (30.3%) presented for initial visit in the first trimester, 90 patients (47.8%) presented initially in the second trimester, and 41 patients (21%) had their first visit in the third trimester. Twenty-one percent of patients had an STD diagnosed at the initial visit. Follow-up STD screening was done according to clinic protocol on all patients at 28 and 36 weeks. This rescreening, performed on all patients and not based upon the presence of symptoms or historical information, revealed that 4.8% had an STD diagnosed at a follow-up visit after the initial STD screen was negative. One percent had a second STD diagnosed at the follow-up visit after the initial screen had already identified one STD. Three percent of the patients had more than one STD.

### STD Characteristics

Twenty-one percent of patients had chlamydia infection [95% confidence interval (CI) = 18.0–24.0%], 4.8% had infection with gonorrhea (95%

CI = 3.3–6.3%), 1.7% new cases of syphilis were identified, and 0.6% had infection with herpes simplex virus. Seven patients tested HIV positive. Sixty-one percent (116/188) of patients with a known STD agreed to HIV testing. One patient had HIV coexistent with another STD (syphilis). The other 6 HIV-positive patients had no coexistent STD.

### DISCUSSION

Pregnant adolescents in our population are at risk for multiple STDs, including HIV. ACOG guidelines recommend STD screening at the initial and follow-up visits. While prenatal care has improved for mothers in all age groups in recent years, teenagers continue to get the least adequate medical attention. A 1978 study reported that mothers under the age of 15 years are 2.5 times more likely than mothers aged 20–24 years to be without prenatal care in the first trimester and nearly 4 times more likely to receive no care or to delay care until the third trimester.[4] Our studies confirm these trends. Because more than half of adolescents presented for the first visit later than the first trimester and because more than 20% of patients had an STD, a scheduled follow-up visit 1–2 weeks after initial presentation and emphatic encouragement regarding compliance with follow-up visits are in order. Repeated testing for STDs will identify those adolescents who continue to test positive because of noncompliance with therapy or continued exposure to the untreated asymptomatic partner. Almost 5% of patients had an STD diagnosed on repeat screening after the initial screen was negative.

The choice of an individual partner and whether that partner resides in an area where the prevalence of STDs is high both contribute to an adolescent's risk.[13,14] An understanding of the adolescent's reasoning process, her concept of monogamy, and knowledge of adolescent sexuality patterns, including those of the male partner, will provide additional areas for intervention.

The attempt to control disease transmission in pregnant adolescents is additionally impaired by the fact that a significant number of teenagers have never used a contraceptive method. Already pregnant teenagers may not understand the rationale for the use of a barrier contraceptive during their pregnancy. In communities where the prevalence of STDs including HIV is high, all pregnant adolescents should be encouraged to use condoms if they remain sexually active during their pregnancy.

The seroprevalence of HIV in adolescents in our community is unknown. There are no reported studies that have reviewed the epidemiology of STDs including HIV in pregnant inner-city adolescents. We identified 7 HIV positive adolescents. None of these patients had obvious risk factors for HIV infection and thus may have acquired the infection through heterosexual contact. Since not all patients agreed to testing, the actual seroprevalence rate among pregnant adolescents in our community (at least 1%) is likely to be higher. The risk and possibility for heterosexual transmission of HIV in this patient group cannot be minimized. While others have suggested that the presence of an STD is a sufficient factor upon which to base decisions regarding HIV screening in adolescents, we noted that only 1 of the 7 patients who tested HIV positive had a coexistent STD. While it is true that 39% of our patients who had STDs did not agree to HIV testing, the discovery of 6 HIV positive patients among the group of patients who tested negative for STDs makes the presence of an STD an insufficient risk factor upon which to base decisions regarding HIV screening in our patient group. We recommend the availability of HIV counseling and screening to all pregnant adolescents, particularly those who reside in communities where the seroprevalence of the disease is high.

While many programs have been developed to meet the needs of pregnant women who test HIV positive, specific programs that address the needs of such infected adolescents are lacking. Ethical and legal issues regarding testing and informing partners, emotional differences in coping mechanisms, and age-related differences in cognitive function make the diagnosis and treatment of HIV infection different in the adolescent than in the adult. While unified support groups specific for subsets of the adult population such as homosexuals, hemophiliacs, and substance abusers exist, few such groups are available to adolescents. The development of multidisciplinary teams with specific attention to the emotional and social needs of HIV-infected adolescents will hopefully assure better perinatal outcomes.

### REFERENCES

1. Centers for Disease Control: Premarital sexual experience among adolescent women—United States 1970–1988. Morbidity Mortality Weekly 39:929–931, 1991.
2. Alan Guttmacher Institute: Teen Sexual and Reproductive Behavior in the U.S. Facts in Brief. New York: Alan Guttmacher Institute, 1990.
3. Alan Guttmacher Institute: Teenage Pregnancy: The Problem That Hasn't Gone Away. New York: Alan Guttmacher Institute, 1981.
4. McAnarney ER: Young age and adverse neonatal outcome. American Journal of Diseases of Children 141: 1053–1059, 1987.
5. Felice M, Grandos JL, Ances IG, et al.: The young pregnant teenager: Impact of comprehensive prenatal care. J Adolescent Health Care 1:193–197, 1981.
6. *Chlamydia trachomatis* infections—Policy guidelines for prevention and control. Morbidity Mortality Weekly 34(Suppl):53–74, 1985.
7. Gravett MG, Nelson HP, DeRouen T, Critchlow C, Eschenbach D, Holmes K: Independent associations of bacterial vaginosis and *Chlamydia trachomatis* infection with adverse pregnancy outcome. JAMA 258:1899–1903, 1986.
8. Shafer MA, Sweet RL, Ohm-Smith MJ: Microbiology of the lower genital tract in post-menarchal adolescent girls: Differences by sexual activity, contraception and presence of nonspecific vaginitis. J Pediatr 107:974–981, 1985.
9. Hein K, Marks A, Cohen M: Asymptomatic gonorrhea: Prevalence in a population of urban adolescents. J Pediatr 90:634, 1977.
10. Chacko MR, Lovchik JC: *Chlamydia trachomatis* infection in sexually active adolescents: Prevalence and risk factors. Pediatrics 73:836–840, 1984.
11. Fraser GJ, Rettig PJ, Kaplan DW: Prevalence of cervical *Chlamydia trachomatis* and *Nelsseria gonorrhoeae* in female adolescents. Pediatrics 71:333–336, 1983.
12. Mumford DM, Smith PB, Goldfarb JL: Prevalence of venereal disease in indigent pregnant adolescents. J Reprod Med 19:83, 1977.
13. Aral SO, Soskoline V, Joesoef RM, O'Reilly K: Sex partner recruitment as risk factor for STD: Clustering of risky modes. Sexually Transmitted Dis 18:10–17, 1991.
14. O'Reilly KR, Aral S: Adolescents and sexual behavior. J Adolescent Health Care 6:262–270, 1985.
